# Mineral Intake Status of Community-Dwelling Elderly from Urban and Rural Areas of South Korea: A Cross-Sectional Study Based on Korean National Health and Nutrition Examination Survey, 2013~2016

**DOI:** 10.3390/ijerph17103415

**Published:** 2020-05-14

**Authors:** Ji-Myung Kim, Yun-Jung Bae

**Affiliations:** 1 Food and Nutrition Major, Division of Food Science and Culinary Arts, Shinhan University, Uijeongbu, Gyeonggi 11644, Korea; kjm@shinhan.ac.kr; 2 Major in Food and Nutrition, Division of Food Science and Biotechnology, Korea National University of Transportation, Jeungpyeong 27909, Korea

**Keywords:** calcium, potassium, rural, Korea, elderly

## Abstract

We aimed to evaluate the intake of minerals (calcium, phosphorous, sodium, and potassium) in the urban and rural elderly and explore the adequacy of intake and food sources for each mineral using nationwide big data. The study used data from the Korea National Health and Nutrition Examination Survey (KNHANES) between 2013 and 2016. We analyzed 5292 elderly individuals that were aged 65 years and older (2271 men, 3021 women). Daily calcium, phosphorous, sodium, and potassium intake, and they were analyzed using the 24-h dietary recall method. Additionally, the adequacy of intake and food sources for each mineral was analyzed. Blood triglyceride level was significantly higher in rural elderly than in urban elderly. The intake of calcium and potassium per 1000 kcal of energy intake was significantly lower in the rural elderly, and the proportion of participants with calcium intake below the Estimated Average Requirement was significantly higher in the rural elderly than in the urban elderly. The intake of calcium, phosphorous, and potassium in the rural elderly was lower than that in the urban elderly. These results can be used as basic data when making social and environmental policies for the health of the elderly and when providing targeted dietary education for the management of chronic diseases for the elderly.

## 1. Introduction

The world population continues to grow older, and the proportion of the world’s population over 60 years of age is expected to nearly double from 12% in 2015 to 22% in 2050 [1]. In South Korea, 14.3% of the total population was 65 years old or over in 2018 [2], and there is growing interest in improving the health and quality of life of the elderly to better encounter population aging. Healthy life in the elderly is dependent on their nutritional status, illness, social, psychological, and economic factors [3].

Good nutrition is one of the key considerations of healthy aging [4]. However, previous studies have showed a decrease in food intake in the elderly. The proportion of people with nutritional deficiency, whose energy intake was lower than 75% of Estimated Energy Requirement and intakes of calcium, iron, vitamin A, and vitamin B_2_, were lower than the Estimated Average Requirements (EAR) of respective nutrients, was 14.4%, the third highest, in the age group of 65 and older; 17.6% in the 12–18 age group; and, 16.2% in the 19–29 age group, according to 2018 Korea National Health and Nutrition Examination Survey (KNHANES) [5] There decrease in the food intake of the elderly is attributed to reduced digestive ability, loss of appetite, poor teeth condition, oral health [6,7,8], and other factors, such as dysphagia, diarrhea, depression, drug, and dysfunction [9]. The level of education and income [10], living alone [11], living area [12], and poor neighborhood food environment [13] are also reported to affect the nutritional status of the elderly.

Elderly health and nutritional status may be associated with dietary differences resulting from urban–rural differences in lifestyles and environmental factors [12,14,15]. Each elderly individual is a member of a community and lives in relationships with others based on where the person lives. Accordingly, the area where a person lives is expected to act as a factor that influences the diet and life of the elderly. The relationship of urban–rural dwellings with chronic diseases differed as much as the urban and rural living environment. In a cohort study, urban residence was one of the strong predictors of type 2 diabetes [16]. In postmenopausal women, the incidence rate of osteoporosis of postmenopausal women was significantly higher among rural residents than among urban residents [17]. Health behaviors and status of the elderly vary depending on the region of residence. A study reported that the health behaviors and status of the elderly in rural areas are better than those in urban areas, despite the worse socioeconomic conditions of rural communities [18]. 

Several non-Korean studies found that the intake of sugar, fat, and animal protein was higher in urban areas, while traditional meals consisting of natural foods were consumed more in rural areas than in urban areas [19,20,21]. Moreover, in the overall assessment of nutritional status, the proportion of poor nutritional status, as evaluated by Mini Nutritional Assessment (MNA), was higher in the urban elderly than in the rural elderly in France [10]; however, there was no significant difference in malnutrition between urban and rural areas in Poland [14]. In Taiwan, the elderly in rural areas reported to have lower self-rated healthy eating status than the elderly in urban areas [15]. Studies [10,14,15] that reported on the comparison of nutritional status of the elderly between urban–rural areas were mostly based on nutritional screening indicators, such as MNA, although a few previous studies have reported comparison of the actual nutrient intake [22].

Studies in Korea showed significantly higher intake of carbohydrate and sodium among the rural adults that were 40–70 years of age [23], while urban people consumed more sugar-sweetened beverages and fast-food [24]. Studies in the elderly showed westernized and meat-based eating habits among the urban elderly, while the elderly in rural areas had healthier eating habits [25]. Yet, a study found that elderly women in the urban areas had significantly higher quality of nutrient intake than rural dwellers [26]. As such, differences in nutrients and food intake between urban and rural areas were observed in the Korean elderly, but the study results were inconsistent. 

Adequate intake of nutrients is suggested for nutritional health to promote healthy aging [27]. The adequate intake of micronutrients (calcium, phosphorous, sodium, and potassium) is closely related to the development of chronic diseases, including hypertension, coronary heart disease, and osteoporosis. Calcium is considered to be a vital nutrient for prevention and management of osteoporosis, obesity, and hypertension [28,29]. Particularly, adequate calcium intake is essential for health maintenance of the elderly when considering adipose tissue accumulation, bone mass loss, and degeneration of blood vessels with aging [30]. Phosphorus deficiency is often observed in patients with prolonged treatment for certain chronic diseases. If elderly without illness are malnourished or are on medications that bind anions, they might suffer from phosphorus depletion [31]. Moreover, the intake of calcium and phosphorus in an appropriate ratio is crucial for the elderly who are at risk of osteoporosis. As the worldwide prevalence of hypertension due to excessive sodium intake continues to be a persistent problem [32], it is very important to maintain proper nutritional status of sodium and potassium in the elderly who are at risk of hypertension that is associated with stiffened blood vessels with aging. Mineral intake in the elderly is associated with their health indicators, as shown in previous studies [30,31]; however, few studies, to date, have compared health indicators between urban and rural elderly and the association between mineral intake and health indicators by regions. 

Mineral intake status of the Korean elderly indicated that the mean calcium intake of the elderly aged 65 years and older was 60% of the Recommended Nutrient Intake (RNI) of calcium, while the mean sodium intake of the same group was 138.2% of the Goal Intake (GI) of sodium (2000 mg) as maximum threshold, thus posing as a prominent risk factor [5]. Additionally, only few studies have focused on the evaluation of mineral intake in the Korean urban and rural elderly, where differences in food intake and some nutrient intake have been reported [22]. Lee et al. [22] reported no significant urban–rural differences in calcium, sodium, and potassium intake after analysis of each mineral based on the RNI. However, another previous study has noted that nutrient intake in the elderly varied depending on the location and gender effect [20]; it is assumed that mineral intake is supposedly different between men and women and between urban and rural settings. We focused on performing an in-depth study on the differences in health status and mineral intake in elderly people, particularly between those living in urban and rural areas, noting the importance of adequate mineral intake in maintaining the health of elderly people. Accordingly, the following strategies were implemented. First, we planned to assess the differences in health indicators between elderly people living in urban and rural areas for promoting their health. Second, we intended to evaluate the adequacy of mineral intake in elderly people living in urban and rural areas to identify minerals that are inadequately consumed in this population. Third, we sought to identify food sources, when considering the unique eating patterns in the Korean population, which contribute to mineral intake to provide basic data for future interventions targeting elderly people living in urban and rural areas with respect to adequate mineral intake. Additional research on the differences in food sources of mineral intake between urban and rural elderly is necessary due to the inconsistencies in results obtained and the unique dietary patterns in Korea. In addition, studies on the comparison of health indicators in urban–rural elderly and the relationship between mineral intake and health indicators are needed. Therefore, we aimed to evaluate the intake of minerals (calcium, phosphorous, sodium, and potassium) in the urban and rural elderly and explore the adequacy of intake and food sources for each mineral while using nationwide big data. 

## 2. Methods

### 2.1. Study Design and Participants

This was a cross-sectional study that analyzed data that were obtained from participants of the KNHANES VI (2013–2015) and VII-1 (2016) conducted from January 2013 to December 2016 by the Korea Centers for Disease Control and Prevention [33]. The KNHANES VI (2013–2015) and VII-1 (2016) was a nationwide, representative survey of 31,098 people from 13,004 households. Survey participants were randomly selected while using a multistage clustered and stratified random sampling method with proportional allocation based on geographic area, sex, and age group from the National Census Data. Survey design and data collection procedures are available elsewhere [34]. The KNHANES consisted of three sections: a health and behavior interview, a health examination, and a dietary survey. We excluded participants who did not respond to dietary surveys (*n* = 3385), those under 65 years (*n* = 22,233), and those who reported implausible daily energy intake (<800 or >4000 kcal/day in men, <500 or >3500 kcal/day in women) (*n* = 188) [35]. Subsequently, a total of 5292 participants (2271 men and 3021 women) were analyzed in the present study. The Institutional Review Board of the Korea Centers for Disease Control and Prevention (2013-07CON-03-4C, 2013-12 EXP-03-5C) for the years 2013 and 2014 approved this study, as the 2015 KNHANES was exempted from research ethics approval based on the Bioethics and Safety Act. Written informed consent was obtained from all of the study participants.

### 2.2. Data Collection

Socio-demographic factors, such as age, sex, living area, education, income, marital status, nutrition education, and residence, were collected using a self-reported general questionnaire. The smoking status and alcohol consumption data were collected while using health and behavior interview. Living area of the aged was classified into two groups: urban and rural. Household income was classified into four groups: low, middle-low, middle-high, and high. The educational level was determined by three categories in accordance with their graduation status (either elementary school, middle school, or high school or higher). Marital status was categorized by married or other, including single, separated, divorced, widowed, and cohabiting. Smoking status was classified as current or past/never. Alcohol intake was determined by four categories in accordance with their frequency of alcohol consumption (never, ≤1/month, 2–4/month, ≥2/week). Nutrition education was classified as “yes” or “no”.

Anthropometric data, blood pressure, and blood profiles were collected while using health examination. Body mass index (BMI) was calculated as weight (kg) divided by height (m) squared (kg/m^2^). Participants were classified into three categories based on BMI (kg/m^2^): <23, 23 ≤ BMI >25, or ≥25. Blood pressure was measured three times using a mercury sphygmomanometer (Baumanometer; Baum Co., Copiague, NY, USA). Using fasting blood samples, total cholesterol, HDL-cholesterol, triglyceride, and glucose were directly measured using a Hitachi Automatic Analyzer 7600 (Hitachi, Tokyo, Japan). LDL-C was calculated using Friedewald’s equation for participants with no data on LDL-C [36]. Hemoglobin was directly measured using a XE-2100D in 2013–2014 and using a XN-9000 in 2015–2016 reports (Sysmex, Kobe, Japan).

Dietary intake data were obtained using a 24-h dietary recall protocol as part of the dietary survey. The dietary data were collected by trained dietitians at the participant’s home, one week after the health interview survey and health examination survey were completed. The total energy and nutrient intake were calculated while using a food composition table that was published by the Rural Development Administration of Korea [37]. The 2015 Dietary Reference Intakes (DRI) for Koreans [38] was used to determine energy and mineral intake status. Macronutrient intake was evaluated as a percentage of total energy intake (% kcal). 

### 2.3. Outcome Measure

Nutrient density, nutrient amount per 1000 kcal, was calculated for calcium, phosphorous, potassium, and sodium. The mineral intake levels were determined by three categories; <75%, 75–125%, or >125% based on the Recommended Nutrient Intake (RNI) for calcium and phosphorus and Adequate Intake (AI) for potassium: Prevalence of insufficient intake was calculated for calcium, phosphorous, and potassium. Insufficient intake was deduced if the mineral intake level was less than the corresponding EAR and RNI for calcium and phosphorus or AI for potassium. The prevalence of excessive intake of sodium was calculated by comparing with GI of 2000 mg. The ratio of calcium to phosphorus was calculated as calcium intake divided by phosphorus intake. The tertiary food codes of the KNHANES were used to identify major food sources of minerals. Food sources of minerals were identified based on the mineral content in each food. The major food sources were determined by taking the ratio of minerals provided by each food over the total intake of mineral from all foods. 

### 2.4. Statistical Analysis

All of the statistical analyses were performed while using the SAS software version 9.4 (SAS Institute, Cary, NC, USA). All of the analyses accounted for the complex survey design, which consisted of multistage, stratified, and clustered samples and survey weights to reflect the estimates of the entire Korean population and avoid biased results. The participants were categorized into two groups, depending on the living area: (1) urban group and (2) rural group. The analyses were separately conducted for men, women, and the total participants. The intake of total energy, macronutrients, and minerals was expressed as mean ± standard error and the food sources of mineral were presented with percentages and cumulative percentages of total consumption. Categorical variables, including age, education, income, marital status, alcohol intake, smoking, nutrition education, BMI categories, and mineral intake categories, were expressed as frequencies and percentages and then compared between the urban and rural groups while using the chi-square test. The blood profiles and intake of minerals by living area was analyzed using the general linear model after adjustment for age, household income level, education level, and total energy intake. The total participants were additionally adjusted for sex variable. *p*-values < 0.05 were considered to be significant.

## 3. Results

Table 1 shows the general characteristics of the participants. The mean age was significantly higher in the rural elderly than in the urban elderly (*p* < 0.001 in men; in women; and, in the total), and the proportion of population over 75 years old was significantly higher in rural areas than in urban areas (*p* = 0.001 in men; *p* < 0.001 in women; and, in the total). The rural participants had significantly lower rates of education and lower household incomes (*p* < 0.001 in men, women, and in the total sample for both the categories). The rural elderly had a larger proportion of participants who were non-drinkers as compared to the urban elderly (*p* = 0.002 in men; *p* = 0.001 in the total), but not women. The proportion of underweight was higher in rural elderly than urban elderly (*p* = 0.040 in the total).

Table 2 shows the participants’ blood pressure and blood profiles. The blood triglyceride level was significantly higher in rural elderly than in urban elderly (*p* = 0.022 in women; *p* = 0.026 in the total), but not men. However, HDL-cholesterol level was significantly lower in rural elderly than in urban elderly only in women (*p* = 0.023).

Table 3 shows the participants’ daily nutrient intake. The total daily energy intakes of total participants were 1665.4 kcal for urban participants and 1688.0 kcal for rural participants. The energy intake of the rural elderly was significantly higher than that of the urban elderly (*p* = 0.002 in men; *p* = 0.001 in the total), but not among women. With respect to the distribution of energy intake of macronutrients, the rural elderly had a significantly higher carbohydrate intake (*p* < 0.001 in men, in women and in the total) and lower protein intake (*p* = 0.002 in women and in the total) and fat intake (*p* < 0.001 in men, in women and in the total) than the urban elderly; however, the proportion of protein intake of men had no significant difference between urban and rural elderly. The intake and nutritional density of the minerals of study showed significant difference among the urban and rural participants; calcium (*p* = 0.001 and *p* < 0.001 in men; *p* = 0.045 and *p* = 0.035 in women; *p* = 0.001 for both intake and nutritional density in the total), phosphorus (*p* = 0.010 and *p* = 0.002 in men; *p* < 0.001 for both intake and nutritional density in women and in the total), and potassium (*p* = 0.043 and *p* = 0.035 in men; *p* = 0.034 and *p* = 0.007 in women; *p* = 0.010 and *p* = 0.003 in the total) with rural elderly consumption being significantly lower than those of the urban elderly. Animal calcium intake (*p* = 0.017 in men; *p* = 0.005 in the total) and the percentage of animal calcium intake (*p* = 0.008 in women and in the total) in the rural elderly were also significantly lower than those in the urban elderly. The calcium to phosphorus ratios in the rural elderly men were significantly lower than those in the urban elderly men (*p* = 0.015).

Table 4 shows the mineral intake levels. The proportions of RNI and below EAR for calcium (*p* = 0.001 and *p* = 0.003 in men; *p* = 0.045 and *p* = 0.033 in women; *p* = 0.001 for both RNI and EAR in the total) and phosphorus (*p* = 0.010 and *p* = 0.021 in men; *p* < 0.001 and *p* = 0.001 in women; *p* < 0.001 and *p* = 0.001 in the total) were significantly lower in the rural elderly than in the urban elderly. Insufficient consumptions less than 75% of RNI of calcium (*p* = 0.011 in men and in the total) and of phosphorus (*p* < 0.001 in women; *p* = 0.001 in the total) were higher in the rural elderly than in the urban elderly. In particular, the proportion of below EAR of calcium was above 80%, and the intake levels of calcium were under 55% of RNI in the total population and only 41.3% and 45.2% of RNI in the rural and urban elderly women, respectively. The proportion of AI for potassium was significantly lower (*p* = 0.043 in men; *p* = 0.034 in women; *p* = 0.010 in the total) and insufficient consumption less than 75% of AI of potassium was higher in the rural elderly than in the urban elderly (*p* = 0.029 in men; *p* = 0.001 in women; *p* < 0.001 in the total). In particular, in the elderly women, the potassium intake was 64.1% of AI in the rural and 69.6% of AI in the urban. 

Table 5 shows the main food sources of minerals. The food sources that provided the most calcium, in descending order, were Chinese cabbage kimchi, milk, and anchovy in Korean rural and urban elderly. The food item that contributed the most to daily calcium intake was Chinese cabbage kimchi for both rural (11.5%) and urban (9.6%) Korean elderly. However, there was difference in the food item that provided the most calcium for the Korean elderly women: Chinese cabbage kimchi (10.1%), rural; milk (11.2%), urban. The percentage of calcium intake that was derived from milk was lower in the Korean rural elderly (6.3%) than in the Korean urban elderly (9.2%). The food sources that provided the most phosphorus were white rice (22.3%), soybean (4.1%), and Chinese cabbage kimchi (3.1%) for the Korean rural elderly and white rice (17.1%), soybean (4.6%), and milk (3.7%) for the Korean urban elderly. The food sources that provided the most potassium were white rice (14.2%), Chinese cabbage kimchi (5.1%), and oriental melon (4.5%) for the Korean rural elderly and white rice (10.8%), Chinese cabbage kimchi (4.4%), and sweet potato (3.7%) for the Korean urban elderly. The food item that contributed the most to daily intake of phosphorus and potassium was white rice. The percentages of phosphorus and potassium intake that were derived from white rice were higher in Korean rural participants than in Korean urban participants. The food sources that provided the most sodium were salt (17.3%), doenjang (14.1%), and Chinese cabbage kimchi (13.1%) for the Korean rural elderly and salt (17.9%), Chinese cabbage kimchi (12.3%), and doenjang (12.3%) for the Korean urban elderly.

## 4. Discussion

There are urban–rural differences in demographic characteristics, income level, and education level that possibly lead to disparities in food supplies and availability and accessibility of foods [20,22,39,40]. The food availability and accessibility may affect the individual’s food choices and eating behaviors and are closely related to the quality of meals served and individual’s nutritional status [41]. Food intake gradually declines with age [5,7,8]. Decreasing food intake of the elderly is related to the deterioration of nutrient intake, specifically insufficient intake of micronutrients. In addition, the nutrient intake of elderly between urban and rural areas displayed a different pattern. A previous study with 1239 Korean elderly aged 65 years and older found that the intake of iron, vitamin A, niacin, and vitamin C among the urban residing elderly was higher than that among the rural residing elderly [22]. Based on these previous findings, in this study, we assumed that there might be differences in mineral intake between the urban and rural elderly. Our results are consistent with the previous findings that the intake of calcium, phosphorus, and potassium was lower in the rural elderly than in the urban elderly and that the proportion of participants with mineral intake below the EAR was significantly higher in the rural elderly than in the urban elderly. Furthermore, primary food sources of calcium, phosphorus, sodium, and potassium were different between both groups.

The mean age of participants in this study was significantly higher in the rural elderly than in urban elderly. The proportion of population aged 65 and older of the total Korean population was 14.3%, according to the 2018 survey [2]. However, the proportion of population aged 65 and older of the total farm household population was 44.7% in Korea, showing a continuous upward trend of population aging in rural areas [42]. Other previous studies also reported that the mean age in rural areas was significantly higher than that in urban areas [23,25]; this is probably due to the rapid industrialization and continued migration of young adults to the cities, resulting in the increased ratio of older adults in rural areas.

Education level is one of the most frequently used indicators of socioeconomic status and it is closely associated with other indicators of socioeconomic status [43]. Lower educational attainment might lead to lower income, which might influence food choice. Rural residents are more likely to have lower education level, a greater proportion of individuals with poverty level household income, and lower accessibility to supermarkets than urban residents [44]. A study reported that the rural elderly had poor access to grocery stores due to transportation difficulties as public transportation is lacking in rural settings [45]. Earlier studies on the urban and rural elderly have reported that both income and education levels of the rural elderly were significantly lower than those of the urban elderly [10,15,20], which is consistent with our findings. Differences in the income and education levels between the urban and rural elderly in the present study were thought to affect food choice and food intake.

This study performed an analysis on the general health indicators in the urban and rural elderly and found significantly lower blood triglyceride levels in urban elderly than in rural elderly. In a study that was conducted in some regions of Indonesia [20], there was no significant difference in hemoglobin levels between urban and rural elderly, but the systolic blood pressure was significantly higher in the rural elderly population than in the urban elderly population. In the study conducted by Chun et al. [18], an analysis was conducted on the relationship between chronic diseases in Korean elderly and residential areas, classified into large cities, cities, and rural areas. In that study, the hypertension diagnosis rate showed no significant difference between the regions, whereas the diabetes diagnosis rate was significantly lower in rural areas. Another previous study reported that a high-carbohydrate diet in Korea was associated with increased triglycerides levels in blood [46]. The rural elderly in this study also showed significantly higher energy intake from carbohydrates than the urban elderly did, which we concluded have caused the high blood triglyceride levels in the rural elderly. Therefore, our findings suggest an urgent need for the development of education programs and measures to achieve recommended carbohydrate intakes in order to prevent dyslipidemia and coronary artery disease in the rural elderly. 

In this study, the energy intake ratio of carbohydrate, protein, and fat was 72.8%, 13.2%, and 13.9% in the urban elderly and 75.8%, 12.6%, and 11.6% in the rural elderly, respectively. A recommended ratio of carbohydrate, protein, and fat was 55~65%, 7~20%, and 15~30%, respectively, according to Acceptable Macronutrient Distribution Ranges in the 2015 DRI for Koreans (KDRIs) [38]. This study’s carbohydrate intake ratio of participants was high when compared to those recommendations, while the protein and fat intake ratio was low. Rural men and women residents had significantly higher energy intake ratio of carbohydrate, but significantly lower energy intake ratio of fat than urban residents. Koreans have a traditional diet consisting of rice as staple food, soup, and side dishes, and, thus, carbohydrates account for a high percentage of Korean meals. Previous studies found that despite the Westernization of Korean diets, the rural elderly tend to maintain traditional Korean diets consisting of carbohydrate-rich foods than the urban elderly [25]. An excessive intake of carbohydrates has been reported as one of the major causes of exacerbation of glucose intolerance [47], and carbohydrate-dense diets are also known to be strongly associated with disorders of lipid metabolism [48]. Therefore, nutrition education on the risk of excessive carbohydrate intake and on the importance of dietary diversity is suggested for the rural elderly. Further research is required on the comprehensive analysis of foods and factors that contribute to carbohydrate intake.

Differences in urban–rural environments and socioeconomic factors may lead to differences in the types of food consumed between regions. According to a previous study, urban elderly residents consumed meats, eggs, fish, milk, fruit, and fast foods, including fatty foods, ice cream, cakes, snacks, and carbonated drinks, but the rural elderly residents consumed more vegetables than the urban elderly in Korea [25]. Such quantitative and qualitative differences in food intake between urban and rural settings eventually lead to disparities in nutrient intake. 

In this study, the proportion of participants that were aged 65 and older that consumed calcium below the EAR was 76.0% in men, 85.2% in women, respectively, showing seriously inadequate calcium intake, which was more severe among elderly women (data not shown). The present study results showed that the intake of calcium, as one of the trace minerals, was remarkably different between the urban and rural elderly men and women, and the proportion of participants with calcium intake below the EAR was significantly higher in rural areas than in urban areas. As age increases, rapid bone loss occurs, which emphasizes the need for calcium nutrition in the elderly. In a previous study, the incidence rate of osteoporosis of postmenopausal women was significantly higher among rural residents than among urban residents [17]. The deficiency of calcium intake in the elderly has been continuously reported in Korea, and the low calcium intake is partly attributed to the relatively limited food sources of calcium [5]. The calcium intake ratio supplied by milk was 7.3% in the urban elderly men and 5.5% in the rural elderly men, according to the analysis of food sources of calcium in this study. The primary food sources of calcium also varied by region among the elderly women. It was milk (11.2%) in urban elderly residents, but it was Chinese cabbage kimchi (10.1%) in rural elderly residents. The urban residents reported high intake of milk, which is a calcium-rich food source with a high absorption rate. The intake ratio of calcium to phosphorus in the urban elderly men (0.45:1) was significantly higher than that in the rural elderly men (0.43:1). The higher calcium intake from milk in the urban elderly than in the rural elderly might be explained by socioeconomic factors. Unlike rice or vegetables, milk needs to be purchased from stores in most cases, because home production of milk is very challenging. However, accessibility to grocery stores for food purchase is lower in rural areas than in urban areas [44], possibly resulting in relatively low calcium intake from milk in the rural elderly. These differences in calcium intake and food sources of calcium between the urban and rural elderly are also expected to affect the onset and management of chronic diseases in the elderly. 

Phosphorus is the second largest mineral in the human body after calcium and is rich in animal foods, such as meat, fish, eggs, and milk, and also in plant sources, such as nuts, vegetables, grains, and tofu. Food sources of phosphorus are abundant in Korean diet and, therefore, the percentage of participants who consumed phosphorus below the EAR was 17.9%, which was lower than that of calcium at 67.4% [5]. In the present study, phosphorus intake was significantly higher in the urban elderly than in the rural elderly, especially the ratio of women rural residents who consumed phosphorus and calcium below the EAR was 40.8% and 88.1%, respectively. Hence, it is necessary to prepare plans to promote adequate intake of calcium and phosphorus in rural elderly women. In this study, supplementary analysis was performed on the each mineral intake and the risk ratio of chronic diseases in urban and rural elderly populations (see Tables S1–S4) to discover that the calcium and phosphorus intake significantly lowered the risk of hypercholesterolemia in elderly men living in urban areas. Additionally, phosphorus intake lowered the risk of hyperglycemia and, thus, homeostasis control of calcium and phosphorus is considered to be important. Calcium combines with fatty acids and bile in the small intestine to inhibit fat absorption, indicating calcium’s effect on lowering cholesterol levels in blood [49]. Based on previous studies, this study presented a negative correlation between calcium intake and hypercholesterolemia; however, the results were inconsistent between urban and rural areas. Along with personal characteristics, urban–rural differences in factors, such as food consumption behavior, food accessibility, and the level of health-nutrition services, lead to differences in the relationship between dietary intake and chronic diseases in urban and rural areas. Therefore, considerably, more systematic studies are needed on the relationship between chronic diseases and calcium and phosphorous intake in urban and rural elderly populations in addition to further investigations of detailed components affecting urban and rural health and dietary intakes.

Potassium has been reported to be protective against hypotension in several epidemiological and clinical studies [50], and white rice, vegetable, kimchi, and fruits are the main sources of potassium in Korea [51]. In this study, the intake of potassium per 1000 kcal of energy intake was significantly higher in the urban elderly than in the rural elderly. This finding is consistent with a large epidemiological study performed in Korea [23] among 40–64-year-old adults. A Polish study also showed that urban inhabitants tended to have higher potassium intake than rural inhabitants [52]. The analysis of potassium food sources in the present study revealed that the cumulative percentage of potassium intake from a total of 10 foods was lower in the urban elderly than in the rural elderly, which implies that the urban elderly consume potassium from more diverse sources than the rural elderly. A recent study on participants aged 50 and older having low calcium intake suggested that potassium intake is beneficial to skeletal health [53], and another previous study revealed that lack of potassium intake was linked to an increased risk of hypertension [54]. Potassium is helpful in preventing chronic diseases, such as hypertension and osteoporosis. However, the analysis on gender differences in this study showed that potassium intake rate as compared to AI was 85.2% in elderly men aged 65 years and older, but 68.2% in women of the same age range, indicating inadequate potassium intake of elderly women (data not shown). In the rural elderly women, the potassium intake was only 64.1% of AI, while sodium intake was 134.3% of GI, showing inappropriate ratio. Additionally, the rural elderly had lower potassium intake and also had limited food sources of potassium compared to the urban elderly. Hence, it is necessary to develop plans to increase the potassium intake among the rural elderly in the future. It is important to note the limitations of this study. First, this study used the 24-h recall method to investigate nutrient intake. This study used raw data from KNHANES, a national nutrition survey, and the shortcomings of a 24-h recall method in the raw data are also applicable to this study. Nevertheless, a stratified and clustered sampling method used for the KNHANES sample design ensured the enhanced representativeness of sample population. Second, the present study was a cross-sectional study and there were difficulties in interpreting causal relationships. Third, the study did not evaluate mineral intake from drinking water due to the characteristic of KNHANES data. The KNHANES surveyed water intake as a whole, including pure water and some teas, such as barley tea and corn tea, which Koreans often drink in lieu of pure water [33]. Thus, further assessment on the mineral intake from drinking water is required in future studies. Fourth, this study analyzed mineral intakes of the Korean elderly and, thus, it is thought to be difficult to generalize the study results to the world population. Fifth, several less-salient factors regarding food purchasing behaviors and food environments were not considered in the study. Differences in nutrients and food sources between urban and rural areas may be closely related to food purchasing environment [55]. However, the KNHANES raw data barely included information regarding food purchasing environment, and this generated limitations in scientific interpretation of research results, if any. 

This study has several strengths at the same time. First, this is the first comprehensive study that systematically analyzed mineral intake of the urban–rural elderly utilizing a national nutrition survey, evaluating intake rate as compared to DRIs, and identifying the food sources of the minerals. Bigger sample population was analyzed than the previous studies to increase statistical power of the present study data [22]. Second, in an effort to eliminate possible biases in the study results, socio-demographic variation, in which differences exist between urban and rural settings, was controlled in the analysis. Third, the previous studies [10,14,15] mostly used nutritional screening indicators for analysis, but this study assessed the actual mineral intake of urban–rural elderly as minerals are essential for elderly health, identifying vital minerals for them, in order to compare nutritional status in the elderly between urban and rural areas. These study results are supposedly of great use as baseline data for nutrition education for health management in rural elderly or for the establishment of health policies. Therefore, further studies are needed to evaluate comprehensive and multi-level variables related to meal intake in the elderly between urban and rural areas. For example, the differences of meal and nutrient intake between the urban and rural elderly would be interpreted in a more systematic way if the number of foods consumed at each meal and the dish-based intake status are known. In addition, a thorough investigation is necessary for food supply and demand variables along with environmental variables related to nutrient intake, especially minerals, among the urban–rural elderly.

## 5. Conclusions

Our study shows that the intake of calcium, phosphorus, and potassium of the rural elderly in Korea was significantly lower than that of the urban elderly. The rural elderly showed more exacerbated status of mineral intake, which resulted in a lower intake ratio as compared to RNI of minerals than the urban elderly did. Additionally, regarding the main food sources contributing to calcium intake and absorption, the rural elderly indicated poor intake of calcium rich food as compared to the urban elderly. Therefore, based on the results of this study, it is necessary to prepare a nutrition policy and implement a nutrition program to increase the mineral intake of rural elderly. Moreover, nutrition education for elderly in rural areas should focus on the mineral intake, such as calcium and potassium, which are necessary for the prevention and management of chronic diseases. Further studies should be carried out to procure more detailed data in order to improve the mineral nutritional status in the urban–rural elderly through the investigation of detailed socio-demographic factors, multifaceted examination of dietary factors, and measurements of clinical biomarkers.

## Figures and Tables

**Table 1:** General characteristics of rural and urban Korean elderly.

		Men (*n* = 2271)			Women (*n* = 3021)			Total (*n* = 5292)		
		Urban (*n* = 1627)	Rural (*n* = 644)	*p* Value	Urban (*n* = 2160)	Rural (*n* = 861)	*p* Value	Urban (*n* = 3787)	Rural (*n* = 1505)	*p* Value
Age (year)		72.0 ± 0.1	73.0 ± 0.2	<0.001	72.6 ± 0.1	73.8 ± 0.2	<0.001	72.4 ± 0.1	73.4 ± 0.2	<0.001
	65–74	1055 (64.8)	382 (59.3)	0.001	1351 (62.5)	449 (52.2)	<0.001	2406 (64.5)	831 (55.1)	<0.001
	≥75	572 (35.2)	262 (40.7)		809 (37.5)	412 (47.9)		1381 (35.5)	674 (44.9)	
Education level (%)	≤Elementary	583 (35.7)	309 (47.2)	<0.001	1429 (65.8)	662 (75.3)	<0.001	2012 (52.4)	971 (63.0)	<0.001
	≤Middle school	254 (15.5)	115 (17.3)		242 (10.9)	46 (5.5)		496 (12.9)	161 (10.6)	
	≥High school	790 (48.8)	220 (35.6)		489 (23.3)	153 (19.2)		1279 (34.7)	373 (26.4)	
Income (%)	Low	631 (38.5)	339 (54.5)	<0.001	1073 (48.8)	547 (65.3)	<0.001	1704 (44.2)	886 (60.6)	<0.001
	Middle-low	487 (28.6)	178 (27.3)		559 (25.2)	184 (20.3)		1046 (26.7)	362 (23.3)	
	Middle-high	282 (18.7)	86 (12.7)		301 (15.5)	74 (8.9)		583 (16.9)	160 (10.6)	
	High	213 (14.2)	36 (5.6)		212 (10.6)	45 (5.5)		425 (12.2)	81 (5.5)	
Marital status (%)	Married	1409 (87.3)	569 (89.0)	0.304	1025 (46.3)	417 (45.2)	0.655	2434 (64.6)	986 (64.3)	0.909
	Others ^(1)^	218 (12.8)	75 (11.0)		1135 (53.7)	444 (54.8)		1353 (35.4)	519 (35.7)	
Alcohol intake (%)	Never	547 (34.8)	241 (38.7)	0.002	1363 (63.8)	579 (69.3)	0.072	1910 (50.9)	820 (56.0)	0.001
	≤1/month	263 (15.9)	96 (16.1)		524 (25.2)	177 (20.7)		787 (21.1)	273 (18.7)	
	2–4/month	303 (19.0)	81 (11.6)		133 (6.3)	47 (5.6)		436 (11.9)	128 (8.1)	
	≥2/week	476 (30.4)	207 (33.7)		97 (4.7)	36 (4.5)		573 (16.1)	243 (17.2)	
Smoking status (%)	Past/never	1229 (79.8)	482 (81.1)	0.560	1950 (97.2)	747 (95.5)	0.191	3179 (89.4)	1229 (89.1)	0.852
	Current	301 (20.2)	111 (18.9)		54 (2.9)	25 (4.5)		355 (10.6)	136 (10.9)	
Nutrition education (%)	Yes	72 (4.2)	31 (3.9)	0.788	121 (5.9)	54 (6.1)	0.808	193 (5.1)	85 (5.2)	0.964
	No	1551 (95.8)	613 (96.1)		2032 (94.1)	804 (93.9)		3583 (94.9)	1417 (94.8)	
Body mass index (kg/m^2^)		23.6 ± 0.1	23.4 ± 0.1	0.151	24.4 ± 0.1	24.2 ± 0.2	0.251	24.0 ± 0.1	23.8 ± 0.1	0.125
	<23	692 (41.2)	292 (46.4)	0.063	740 (34.0)	321 (36.7)	0.311	1432 (37.2)	613 (40.9)	0.040
	23~<25	443 (28.3)	153 (23.9)		549 (25.9)	203 (22.9)		992 (26.9)	356 (23.3)	
	≥25	492 (30.5)	199 (29.7)		871 (40.1)	337 (40.5)		1363 (35.8)	536 (35.8)	

Data represent mean ± standard error or number of case (%). Categorical variables were analyzed using the Chi-square test. Continuing variables were analyzed using the general linear model. Total data was additionally adjusted for sex using Chi-square test for categorical variables and general linear model for continuous variables. ^(1)^ Widowed, separated, divorced, or never married.

**Table 2:** Blood pressure and blood profiles of rural and urban Korean elderly.

	Men (*n* = 2271)			Women (*n* = 3021)			Total (*n* = 5292)		
	Urban (*n* = 1627)	Rural (*n* = 644)	*p* Value	Urban (*n* = 2160)	Rural (*n* = 861)	*p* Value	Urban (*n* = 3787)	Rural (*n* = 1505)	*p* Value
Systolic blood pressure (mmHg)	127.0 ± 0.5	125.4 ± 0.9	0.090	129.8 ± 0.5	129.2 ± 0.8	0.153	128.6 ± 0.2	127.5 ± 0.7	0.051
Diastolic blood pressure (mmHg)	72.3 ± 0.3	70.8 ± 0.6	0.276	72.0 ± 0.3	71.6 ± 0.4	0.927	72.2 ± 0.2	71.3 ± 0.4	0.448
Fasting blood glucose (mg/dL)	109.1 ± 0.9	106.7 ± 1.2	0.200	106.4 ± 0.6	105.6 ± 1.4	0.731	107.7 ± 0.5	106.1 ± 0.9	0.255
Hemoglobin (mg/dL)	14.5 ± 0.1	14.5 ± 0.1	0.385	13.1 ± 0.1	13.0 ± 0.1	0.450	13.7 ± 0.1	13.7 ± 0.1	0.996
Triglyceride (mg/dL)	134.4 ± 2.5	139.0 ± 3.9	0.394	135.6 ± 1.9	149.0 ± 5.2	0.022	135.0 ± 1.6	144.4 ± 3.5	0.026
Total cholesterol (mg/dL)	179.3 ± 1.1	178.2 ± 1.8	0.611	192.0 ± 1.0	194.2 ± 1.7	0.276	186.1 ± 0.8	186.9 ± 1.3	0.646
HDL-cholesterol (mg/dL)	46.4 ± 0.4	46.2 ± 0.5	0.913	49.9 ± 0.3	48.2 ± 0.5	0.023	48.3 ± 0.3	47.3 ± 0.4	0.082
LDL-cholesterol (mg/dL)	198.8 ± 1.3	196.6 ± 2.2	0.450	214.8 ± 1.2	212.6 ± 2.0	0.481	207.4 ± 0.9	205.3 ± 1.5	0.308

Data represent mean ± standard error. Continuous variables were analyzed using the general linear model after adjusting for age, household income level, education level, and total energy intake. Total data was additionally adjusted for sex using general linear model.

**Table 3:** Daily energy, macronutrient, and mineral intake levels of rural and urban Korean elderly.

		Men (*n* = 2271)			Women (*n* = 3021)			Total (*n* = 5292)		
		Urban (*n* = 1627)	Rural (*n* = 644)	*p* Value	Urban (*n* = 2160)	Rural (*n* = 861)	*p* Value	Urban (*n* = 3787)	Rural (*n* = 1505)	*p* Value
Energy (kcal)		1919.5 ± 19.4	1971.8 ± 30.9	0.002	1460.7 ± 14.4	1468.2 ± 23.8	0.057	1665.4 ± 13.2	1688.0 ± 23.0	0.001
Energy distribution	% Carbohydrate	71.5 ± 0.3	73.9 ± 0.4	<0.001	73.9 ± 0.3	77.3 ± 0.4	<0.001	72.8 ± 0.2	75.8 ± 0.4	<0.001
	% Protein	13.8 ± 0.1	13.4 ± 0.2	0.158	12.8 ± 0.1	12.1 ± 0.2	0.002	13.2 ± 0.1	12.6 ± 0.1	0.002
	% Fat	14.7 ± 0.2	12.8 ± 0.3	<0.001	13.3 ± 0.2	10.7 ± 0.3	<0.001	13.9 ± 0.2	11.6 ± 0.3	<0.001
Calcium (mg)		469.0 ± 10.1	421.8 ± 12.6	0.001	361.9 ± 6.1	330.4 ± 10.1	0.045	409.7 ± 6.1	370.3 ± 9.1	0.001
Calcium (mg/1000 kcal)		247.0 ± 4.0	216.9 ± 5.1	<0.001	249.5 ± 3.5	226.3 ± 6.2	0.035	248.3 ± 2.8	222.2 ± 4.8	0.001
Animal calcium (mg)		151.4 ± 7.6	120.7 ± 8.0	0.017	121.1 ± 4.4	99.3 ± 6.7	0.081	134.6 ± 4.3	108.7 ± 5.6	0.005
Animal calcium (%)		26.5 ± 0.6	24.0 ± 0.9	0.265	26.9 ± 0.6	22.9 ± 0.9	0.008	26.7 ± 0.4	23.4 ± 0.7	0.008
Phosphorus (mg)		1018.6 ± 13.5	977.8 ± 21.1	0.010	774.7 ± 9.5	715.1 ± 15.9	<0.001	883.6 ± 9.2	829.8 ± 15.2	<0.001
Phosphorus (mg/1000 kcal)		532.9 ± 4.1	496.2 ± 6.3	0.002	530.4 ± 3.6	485.7 ± 6.6	<0.001	531.5 ± 3.0	490.2 ± 5.5	<0.001
Calcium: Phosphorus ratio		0.45:1	0.43:1	0.015	0.46:1	0.46:1	0.873	0.46:1	0.45:1	0.177
Sodium (mg)		3736.4 ± 69.2	3740.5 ± 119.0	0.723	2625.3 ± 45.2	2686.7 ± 97.2	0.502	3121.0 ± 44.0	3146.8 ± 86.8	0.816
Sodium (mg/1000 kcal)		1969.6 ± 32.5	1929.9 ± 50.5	0.863	1809.8 ± 26.6	1848.0 ± 59.6	0.570	1881.1 ± 21.4	1883.7 ± 45.1	0.738
Potassium (mg)		3013.1 ± 45.5	2876.6 ± 72.1	0.043	2435.2 ± 35.6	2243.8 ± 60.7	0.034	2693.1 ± 32.3	2520.1 ± 56.4	0.010
Potassium (mg/1000 kcal)		1577.5 ± 15.7	1467.4 ± 26.9	0.035	1677.8 ± 18.7	1521.7 ± 32.1	0.007	1633.0 ± 13.6	1498.0 ± 25.8	0.003

Data represent mean ± standard error. *p* values were calculated by general linear model after adjusting for age, household income level, education level, and total energy intake. Total data was additionally adjusted for sex using general linear model.

**Table 4:** Mineral intake levels by Dietary Reference Intakes (DRI) of rural and urban Korean elderly.

		Men (*n* = 2271)			Women (*n* = 3021)			Total (*n* = 5292)		
		Urban (*n* = 1627)	Rural (*n* = 644)	*p* Value	Urban (*n* = 2160)	Rural (*n* = 861)	*p* Value	Urban (*n* = 3787)	Rural (*n* = 1505)	*p* Value
Calcium	RNI (%)	67.0 ± 1.5	60.3 ± 1.8	0.001	45.2 ± 0.8	41.3 ± 1.3	0.045	54.9 ± 0.8	49.6 ± 1.2	0.001
	<75% RNI	1098 (68.1)	489 (75.8)	0.011	1901 (87.5)	778 (89.2)	0.511	2999 (78.9)	1267 (83.4)	0.011
	75–125% RNI	420 (25.8)	125 (19.2)		219 (10.7)	67 (8.9)		639 (17.4)	192 (13.4)	
	>125% RNI	109 (6.1)	30 (5.0)		40 (1.8)	16 (1.9)		149 (3.8)	46 (3.2)	
	<EAR	1199 (74.3)	522 (81.4)	0.003	1829 (84.2)	769 (88.1)	0.033	3028 (79.8)	1291 (85.2)	0.001
Phosphorus	RNI (%)	145.5 ± 1.9	139.7 ± 3.0	0.010	110.7 ± 1.4	102.2 ± 2.3	<0.001	126.2 ± 1.3	118.5 ± 2.2	<0.001
	<75% RNI	126 (8.0)	68 (9.2)	0.264	550 (25.3)	280 (34.0)	<0.001	676 (17.6)	348 (23.2)	0.001
	75–125% RNI	566 (35.3)	238 (38.5)		907 (42.3)	361 (40.7)		1473 (39.1)	599 (39.8)	
	>125% RNI	935 (56.7)	338 (52.3)		703 (32.5)	220 (25.3)		1638 (43.3)	558 (37.1)	
	<EAR	190 (11.8)	109 (16.2)	0.021	714 (33.1)	343 (40.8)	0.001	904 (23.6)	452 (30.1)	0.001
Sodium	GI (%)	186.8 ± 3.5	187.0 ± 6.0	0.723	131.3 ± 2.3	134.3 ± 4.9	0.502	156.1 ± 2.2	157.3 ± 4.3	0.816
	>GI	1297 (79.6)	508 (79.07)	0.789	1236 (56.9)	483 (54.3)	0.285	2533 (67.1)	991 (65.1)	0.322
Potassium	AI (%)	86.1 ± 1.3	82.2 ± 2.1	0.043	69.6 ± 1.0	64.1 ± 1.7	0.034	76.9 ± 0.9	72.0 ± 1.6	0.010
	<75% AI	726 (46.7)	336 (53.3)	0.029	1387 (63.9)	625 (72.5)	0.001	2110 (56.2)	961 (64.1)	<0.001
	75–125% AI	643 (37.9)	214 (31.5)		623 (29.2)	179 (21.1)		1266 (33.1)	393 (25.7)	
	>125% AI	258 (15.4)	94 (15.2)		153 (6.9)	57 (6.4)		411 (10.7)	151 (25.7)	

RNI, recommended nutrient intake; EAR, estimated average requirement; AI, adequate intake; GI, goal intake (2000 mg/day). Data represent mean ± standard error or number of case (%). Categorical variables were analyzed using the Chi-square test. Continuing variables were analyzed using the general linear model after adjusting for age, household income level, education level, and total energy intake. Total data was additionally adjusted for sex using Chi-square test for categorical variables and general linear model for continuous variables.

**Table 5:** Main food sources of minerals of rural and urban Korean elderly.

		Men (*n* = 2271)						Women (*n* = 3021)						Total (*n* = 5292)					
		Urban (*n* = 1627)			Rural (*n* = 644)			Urban (*n* = 2160)			Rural (*n* = 861)			Urban (*n* = 3787)			Rural (*n* = 1505)		
Minerals	Rank	Food name	%	Cum%	Food name	%	Cum%	Food name	%	Cum%	Food name	%	Cum%	Food name	%	Cum%	Food name	%	Cum%
Calcium	1	Kimchi ^(1)^	10.6	10.6	Kimchi ^(1)^	13.0	13.0	Milk	11.2	11.2	Kimchi ^(1)^	10.1	10.1	Kimchi ^(1)^	9.6	9.6	Kimchi ^(1)^	11.5	11.5
	2	Milk	7.3	17.9	Milk	5.5	18.5	Kimchi ^(1)^	8.5	19.7	Milk	7.2	17.3	Milk	9.2	18.8	Milk	6.3	17.8
	3	Anchovy	6.4	24.4	Anchovy	4.9	23.4	Anchovy	5.2	24.9	Anchovy	5.5	22.7	Anchovy	5.8	24.7	Anchovy	5.2	23.0
	4	Soybean	3.7	28.1	White rice	3.9	27.3	Soybean	3.5	28.5	White rice	4.3	27.0	Soybean	3.6	28.3	White rice	4.1	27.1
	5	Pond loach	3.2	31.3	Soybean	3.5	30.8	White rice	3.1	31.5	Soybean	3.1	30.1	White rice	3.0	31.3	Soybean	3.3	30.4
	6	White rice	2.9	34.2	Pond loach	2.7	33.4	Yoghurt, Liquid type	3.0	34.5	Yoghurt, Liquid type	2.6	32.7	Sea mustard	2.7	34.0	Welsh onion	2.4	32.9
	7	Sea mustard	2.8	37.0	Welsh onion	2.5	35.9	Sea mustard	2.7	37.2	Welsh onion	2.4	35.1	Pond loach	2.6	36.6	Sea mustard	2.2	35.1
	8	Tofu	2.3	39.3	Tofu	2.1	38.1	Egg	2.0	39.2	Sea mustard	2.3	37.4	Yoghurt, Liquid type	2.4	39.0	Yoghurt, Liquid type	2.1	37.2
	9	Welsh onion	2.2	41.5	Coffee	2.1	40.2	Pond loach	2.0	41.1	Crab	2.1	39.5	Tofu	2.1	41.1	Pond loach	2.0	39.1
	10	Egg	2.0	43.5	Sea mustard	2.1	42.2	Tofu	1.9	43.0	Doenjang	2.1	41.7	Welsh onion	2.0	43.1	Doenjang	2.0	41.1
Phosphorus	1	White rice	16.5	16.5	White rice	21.0	21.0	White rice	17.9	17.8	White rice	23.7	23.7	White rice	17.1	17.1	White rice	22.3	22.3
	2	Soybean	4.7	21.2	Soybean	4.2	25.2	Soybean	4.6	22.4	Soybean	3.9	27.6	Soybean	4.6	21.8	Soybean	4.1	26.4
	3	Egg	3.2	24.5	Kimchi ^(1)^	3.4	28.6	Milk	4.4	26.8	Kimchi ^(1)^	2.8	30.4	Milk	3.7	25.5	Kimchi ^(1)^	3.1	29.5
	4	Glutinous rice	3.0	27.5	Pork	3.2	31.9	Glutinous rice	3.6	30.4	Brown rice	2.7	33.1	Glutinous rice	3.3	28.8	Brown rice	2.7	32.2
	5	Brown rice	3.0	30.5	Egg	2.7	34.6	Brown rice	3.3	33.6	Milk	2.4	35.5	Egg	3.2	32.0	Pork	2.6	34.8
	6	Kimchi ^(1)^	3.0	33.5	Brown rice	2.7	37.3	Egg	3.2	36.8	Egg	2.4	37.9	Brown rice	3.1	35.1	Egg	2.5	37.4
	7	Milk	3.0	36.4	Tofu	2.6	39.9	Beef	2.5	39.3	Anchovy	2.3	40.2	Kimchi ^(1)^	2.7	37.8	Tofu	2.3	39.7
	8	Tofu	2.9	39.3	Coffee	2.5	42.5	Tofu	2.4	41.7	Glutinous rice	2.3	42.5	Tofu	2.7	40.5	Glutinous rice	2.3	42.0
	9	Pork	2.9	42.2	Beef	2.4	44.9	Kimchi ^(1)^	2.4	44.1	Beef	2.1	44.6	Beef	2.6	43.1	Beef	2.2	44.2
	10	Anchovy	2.9	45.0	Glutinous rice	2.3	47.1	Anchovy	2.3	46.4	Tofu	2.0	46.6	Anchovy	2.6	45.7	Milk	2.2	46.4
Sodium	1	Salt	18.4	18.4	Salt	18.3	18.3	Salt	17.3	17.3	Salt	16.2	16.2	Salt	17.9	17.9	Salt	17.3	17.3
	2	Kimchi ^(1)^	13.2	31.6	Kimchi ^(1)^	14.3	32.5	Doenjang	13.7	31.0	Doenjang	15.8	32.1	Kimchi ^(1)^	12.3	30.2	Doenjang	14.1	31.4
	3	Doenjang	10.9	42.6	Doenjang	12.5	45.0	Kimchi ^(1)^	11.3	42.3	Kimchi ^(1)^	12.0	44.0	Doenjang	12.3	42.4	Kimchi ^(1)^	13.1	44.5
	4	Soy sauce	10.6	53.2	Soy sauce	10.3	55.3	Soy sauce	10.5	52.9	Soy sauce	10.6	54.6	Soy sauce	10.6	53.0	Soy sauce	10.4	55.0
	5	Fermented red pepper paste	3.6	56.8	Noodle	4.2	59.5	Noodle	3.4	56.2	Noodle	4.0	58.6	Noodle	3.3	56.4	Noodle	4.1	59.1
	6	Noodle	3.3	60.1	Ramyeon	3.6	63.1	Fermented red pepper paste	2.6	58.8	Fermented red pepper paste	2.6	61.2	Fermented red pepper paste	3.1	59.5	Fermented red pepper paste	3.0	62.0
	7	Ramyeon	3.0	63.0	Fermented red pepper paste	3.3	66.4	Ramyeon	2.3	61.1	Kimchi, Dongchimi	2.5	63.7	Ramyeon	2.6	62.1	Ramyeon	2.6	64.6
	8	Salt-fermented seafood	2.1	65.2	Salt-fermented seafood	3.0	69.4	Sea mustard	2.2	63.3	Rice cake	2.2	65.9	Sea mustard	2.1	64.2	Salt-fermented seafood	2.5	67.2
	9	Sea mustard	2.1	67.3	Kimchi, Dongchimi	1.8	71.2	Rice cake	2.2	65.4	Kimchi, Yeolmumulkimchi	2.1	68.1	Salt-fermented seafood	2.0	66.2	Kimchi, Nabakkimchi	2.2	69.3
	10	Kimchi, Yeolmumulkimchi,	1.5	68.8	Kimchi, Yeolmumulkimchi	1.6	72.8	Kimchi, Nabakkimchi	2.1	67.6	Salt-fermented seafood	2.0	70.1	Kimchi, Yeolmumulkimchi	1.8	68.0	Kimchi, Yeolmumulkimchi	1.9	71.2
Potassium	1	White rice	10.8	10.8	White rice	13.7	13.7	White rice	10.9	10.9	White rice	14.6	14.6	White rice	10.8	10.8	White rice	14.2	14.2
	2	Kimchi ^(1)^	5.1	15.9	Kimchi ^(1)^	5.8	19.5	Sweet potato	4.3	15.2	Oriental melon	4.8	19.3	Kimchi ^(1)^	4.4	15.3	Kimchi ^(1)^	5.1	19.3
	3	Soybean	3.7	19.5	Oriental melon	4.3	23.8	Oriental melon	4.1	19.2	Kimchi ^(1)^	4.5	23.8	Sweet potato	3.7	19.0	Oriental melon	4.5	23.8
	4	Apple	3.4	23.0	Coffee	3.4	27.3	Kimchi ^(1)^	3.8	23.1	Potato	4.2	28.0	Soybean	3.5	22.5	Potato	3.7	27.5
	5	Sweet potato	3.1	26.0	Soybean	3.2	30.5	Apple	3.4	26.5	Sweet potato	3.6	31.6	Apple	3.4	25.9	Sweet potato	3.2	30.7
	6	Coffee	2.9	28.9	Potato	3.2	33.7	Potato	3.4	29.9	Soybean	2.9	34.5	Oriental melon	3.3	29.2	Soybean	3.1	33.7
	7	Radish	2.9	31.8	Radish	2.9	36.5	Soybean	3.3	33.2	Apple	2.5	37.0	Potato	2.9	32.0	Coffee	2.8	36.6
	8	Oriental melon	2.4	34.2	Sweet potato	2.7	39.2	Radish	2.6	35.8	Radish	2.3	39.3	Radish	2.7	34.7	Radish	2.6	39.1
	9	Potato	2.3	36.5	Hot pepper	2.3	41.5	Milk	2.4	38.2	Coffee	2.3	41.6	Coffee	2.5	37.2	Apple	2.3	41.4
	10	Sea mustard	2.3	38.8	Apple	2.0	43.5	Coffee	2.0	40.3	Persimmon	2.0	43.6	Sea mustard	2.1	39.3	Hot pepper	2.0	43.4

%; Percentages of total intake. Cum%; Cumulative percentages of total intake. ^(1)^ Kimchi, Chinese cabbage kimchi.

## Supplementary Materials

The following are available online at https://www.mdpi.com/1660-4601/17/10/3415/s1. Table S1. Odds ratio of hypertension and mineral intake levels of rural and urban Korean elderly, Table S2. Odds ratio of hypercholesterolemia and mineral intake levels of rural and urban Korean elderly, Table S3. Odds ratio of hyperglycemia and mineral intake levels of rural and urban Korean elderly, Table S4. Odds ratio of hypertriglyceridemia and mineral intake levels of rural and urban Korean elderly.
