# Supplementary material for: Mineral Intake Status of Community-Dwelling Elderly from Urban and Rural Areas of South Korea: A Cross-Sectional Study Based on Korean National Health and Nutrition Examination Survey, 2013~2016
Source: Int J Environ Res Public Health. 2020 May 14;17(10):3415. doi: 10.3390/ijerph17103415 (PMC7277109; doi:10.3390/ijerph17103415)
Supplement: Supplementary file 1 [file ijerph-17-03415-s001.pdf]

Table S1. Odds ratio of hypertension and mineral intake levels of rural and urban Korean elderly

Continuous variables (per daily 10 mg increment)		Men (n=2,271)		Women (n = 3,021)		Total (n=5,292)	
		Urban (n=1,627)	Rural (n=644)	Urban (n=2,160)	Rural (n=861)	Urban (n=3,787)	Rural (n=1,505)
Calcium							
Model I	OR(CI)	1.001(0.997-1.004)	1.002(0.993-1.012)	0.995(0.988-1.002)	0.999(0.990-1.007)	0.998(0.994-1.001)	0.999(0.993-1.005)
	P value	0.790	0.638	0.160	0.737	0.194	0.707
Model II	OR(CI)	1.001(0.997-1.005)	1.004(0.992-1.015)	0.995(0.988-1.003)	1.005(0.993-1.016)	0.999(0.995-1.003)	1.004(0.996-1.012)
	P value	0.672	0.542	0.249	0.423	0.642	0.345
Phosphorus							
Model I	OR(CI)	0.998(0.995-1.002)	1.000(0.995-1.005)	0.998(0.993-1.003)	0.997(0.991-1.002)	0.997(0.995-1.000)	0.997(0.993-1.001)
	P value	0.330	0.875	0.393	0.256	0.043	0.096
Model II	OR(CI)	0.995(0.990-1.001)	1.000(0.993-1.008)	0.998(0.992-1.004)	1.002(0.993-1.012)	0.996(0.992-1.000)	1.001(0.995-1.007)
	P value	0.081	0.918	0.543	0.628	0.071	0.659
Sodium							
Model I	OR(CI)	1.000(1.000-1.001)	1.000(0.999-1.001)	1.000(0.999-1.001)	1.000(0.999-1.001)	1.000(1.000-1.001)	1.000(0.999-1.000)
	P value	0.261	0.538	0.966	0.585	0.816	0.532
Model II	OR(CI)	1.000(1.000-1.001)	1.000(0.999-1.001)	1.000(0.999-1.001)	1.000(0.999-1.001)	1.000(1.000-1.001)	1.000(1.000-1.001)
	P value	0.283	0.428	0.898	0.968	0.270	0.499
Potassium							
Model I	OR(CI)	1.000(0.999-1.000)	1.000(0.999-1.001)	0.999(0.998-1.001)	0.999(0.998-1.000)	0.999(0.999-1.000)	0.999(0.998-1.000)
	P value	0.439	0.660	0.372	0.198	0.073	0.053
Model II	OR(CI)	1.000(0.998-1.001)	0.999(0.997-1.001)	0.999(0.998-1.001)	1.000(0.998-1.002)	1.000(0.999-1.000)	1.000(0.998-1.001)
	P value	0.468	0.290	0.350	0.993	0.225	0.528

Hypertension, blood pressure ≥ 120/80 mmHg or medication; OR, odds ratio; CI, confidence interval; Model I, crude; Model II, age, household income level, education level, and total energy intake adjusted; Total data was additionally adjusted for sex.

Table S2. Odds ratio of hypercholesterolemia and mineral intake levels of rural and urban Korean elderly

Continuous variables		Men (n=2,271)		Women (n = 3,021)		Total (n=5,292)	
(per daily 10 mg increment)		Urban (n=1,627)	Rural (n=644)	Urban (n=2,160)	Rural (n=861)	Urban (n=3,787)	Rural (n=1,505)
Calcium							
Model I	OR(CI)	0.998(0.993-1.002)	1.001(0.991-1.011)	1.003(0.998-1.009)	1.006(0.999-1.014)	0.997(0.994-1.000)	1.001(0.995-1.008)
	P value	0.315	0.859	0.206	0.111	0.097	0.673
Model II	OR(CI)	0.993(0.987-0.999)	1.004(0.991-1.018)	1.002(0.995-1.008)	1.005(0.997-1.014)	0.997(0.993-1.001)	1.005(0.998-1.012)
	P value	0.018	0.544	0.620	0.218	0.168	0.146
Phosphorus							
Model I	OR(CI)	1.000(0.996-1.004)	1.002(0.996-1.008)	1.003(0.999-1.006)	1.000(0.995-1.006)	0.998(0.996-1.000)	0.997(0.993-1.002)
	P value	0.963	0.587	0.116	0.895	0.082	0.202
Model II	OR(CI)	0.994(0.988-0.999)	1.007(0.998-1.017)	1.001(0.996-1.007)	1.000(0.989-1.010)	0.997(0.994-1.001)	1.003(0.997-1.010)
	P value	0.025	0.143	0.664	0.937	0.206	0.338
Sodium							
Model I	OR(CI)	1.001(1.000-1.001)	1.000(0.999-1.001)	1.000(0.999-1.001)	1.000(0.999-1.001)	1.000(0.999-1.000)	0.999(0.999-1.000)
	P value	0.039	0.818	0.835	0.866	0.418	0.116
Model II	OR(CI)	1.000(1.000-1.001)	1.000(0.999-1.001)	1.000(0.999-1.001)	1.000(0.999-1.00)	1.000(1.000-1.001)	1.000(0.999-1.001)
	P value	0.132	0.890	0.810	0.799	0.439	0.911
Potassium							
Model I	OR(CI)	1.000(1.000-1.001)	1.001(0.999-1.003)	1.001(1.000-1.002)	1.000(0.999-1.002)	1.000(0.999-1.001)	1.000(0.999-1.001)
	P value	0.347	0.385	0.069	0.555	0.936	0.888
Model II	OR(CI)	1.000(0.999-1.001)	1.002(0.999-1.004)	1.000(0.999-1.001)	1.001(0.999-1.002)	1.000(0.999-1.001)	1.001(1.000-1.002)
	P value	0.663	0.159	0.511	0.562	0.801	0.193

Hypercholesterolemia, total cholesterol ≥ 240 mg/dL or medication; OR, odds ratio; CI, confidence interval; Model I, crude; Model II, age, household income level, education level, and total energy intake adjusted; Total data was additionally adjusted for sex.

Table S3. Odds ratio of hyperglycemia and mineral intake levels of rural and urban Korean elderly

Continuous variables		Men (n=2,271)		Women (n = 3,021)		Total (n=5,292)	
(per daily 10 mg increment)		Urban (n=1,627)	Rural (n=644)	Urban (n=2,160)	Rural (n=861)	Urban (n=3,787)	Rural (n=1,505)
Calcium							
Model I	OR(CI)	1.002(0.998-1.006)	0.993(0.986-1.000)	0.995(0.990-1.000)	1.001(0.993-1.009)	1.000(0.997-1.003)	0.998(0.993-1.003)
	P value	0.392	0.038	0.049	0.820	0.943	0.429
Model II	OR(CI)	1.003(0.998-1.007)	0.992(0.983-1.000)	0.994(0.988-1.000)	1.003(0.995-1.012)	1.000(0.996-1.004)	0.998(0.992-1.004)
	P value	0.208	0.063	0.061	0.441	0.979	0.508
Phosphorus							
Model I	OR(CI)	1.000(0.997-1.003)	0.997(0.993-1.001)	0.997(0.993-1.000)	0.998(0.992-1.003)	0.999(0.997-1.002)	0.998(0.995-1.001)
	P value	0.971	0.107	0.037	0.393	0.632	0.176
Model II	OR(CI)	1.003(0.998-1.007)	0.996(0.988-1.003)	0.994(0.988-0.999)	0.998(0.989-1.008)	0.999(0.996-1.003)	0.997(0.992-1.002)
	P value	0.245	0.255	0.025	0.676	0.647	0.234
Sodium							
Model I	OR(CI)	1.000(0.999-1.000)	1.000(0.999-1.000)	1.000(0.999-1.001)	1.000(0.998-1.001)	1.000(1.000-1.000)	1.000(0.999-1.000)
	P value	0.368	0.643	0.698	0.499	0.823	0.583
Model II	OR(CI)	1.000(0.999-1.000)	1.000(0.999-1.001)	1.000(0.999-1.001)	1.000(0.999-1.001)	1.000(1.000-1.000)	1.000(0.999-1.001)
	P value	0.579	0.971	0.766	0.671	0.866	0.853
Potassium							
Model I	OR(CI)	1.000(0.999-1.001)	0.999(0.998-1.001)	0.999(0.998-1.000)	0.999(0.998-1.001)	1.000(0.999-1.000)	1.000(0.999-1.001)
	P value	0.895	0.424	0.019	0.438	0.241	0.408
Model II	OR(CI)	1.000(0.999-1.001)	1.000(0.998-1.001)	0.999(0.998-1.000)	1.000(0.998-1.001)	1.000(0.999-1.000)	1.000(0.999-1.001)
	P value	0.490	0.630	0.044	0.813	0.312	0.617

Hyperglycemia, fasting glucose ≥ 100 mg/dL or medication; OR, odds ratio; CI, confidence interval; Model I, crude; Model II, age, household income level, education level, and total energy intake adjusted; Total data was additionally adjusted for sex.

Table S4. Odds ratio of hypertriglyceridemia and mineral intake levels of rural and urban Korean elderly

Continuous variables (per daily 10 mg increment)		Men (n=2,271) Urban (n=1,627)	Men (n=2,271) Rural (n=644)	Women (n = 3,021) Urban (n=2,160)	Women (n = 3,021) Rural (n=861)	Total (n=5,292) Urban (n=3,787)	Total (n=5,292) Rural (n=1,505)
Calcium							
Model I	OR(CI)	0.999(0.993-1.005)	1.007(0.993-1.020)	0.994(0.987-1.001)	1.006(0.994-1.019)	0.997(0.992-1.003)	1.006(0.997-1.014)
	P value	0.744	0.323	0.094	0.317	0.346	0.175
Model II	OR(CI)	1.001(0.994-1.007)	1.010(0.993-1.027)	0.995(0.985-1.004)	1.008(0.992-1.023)	0.999(0.993-1.005)	1.009(0.998-1.019)
	P value	0.867	0.248	0.260	0.338	0.712	0.110
Phosphorus							
Model I	OR(CI)	0.997(0.993-1.001)	1.001(0.994-1.008)	0.997(0.992-1.002)	1.001(0.993-1.008)	0.997(0.994-1.000)	1.000(0.995-1.005)
	P value	0.170	0.747	0.213	0.851	0.097	0.918
Model II	OR(CI)	0.998(0.992-1.005)	1.003(0.991-1.015)	0.997(0.990-1.005)	1.001(0.987-1.014)	0.998(0.993-1.003)	1.002(0.993-1.010)
	P value	0.654	0.604	0.512	0.940	0.444	0.673
Sodium							
Model I	OR(CI)	1.000(0.999-1.001)	1.000(0.999-1.001)	1.000(0.999-1.001)	0.999(0.998-1.000)	1.000(1.000-1.001)	0.999(0.999-1.000)
	P value	0.889	0.566	0.488	0.250	0.538	0.202
Model II	OR(CI)	1.000(1.000-1.001)	1.000(0.999-1.001)	1.001(1.000-1.002)	0.999(0.998-1.001)	1.000(1.000-1.001)	0.999(0.998-1.000)
	P value	0.357	0.623	0.129	0.205	0.112	0.204
Potassium							
Model I	OR(CI)	0.999(0.998-1.000)	1.000(0.998-1.002)	1.000(0.998-1.001)	1.001(0.999-1.002)	0.999(0.999-1.000)	1.000(0.999-1.001)
	P value	0.180	0.782	0.488	0.354	0.156	0.801
Model II	OR(CI)	1.000(0.998-1.001)	1.000(0.997-1.002)	1.000(0.999-1.001)	1.001(0.999-1.002)	1.000(0.999-1.001)	1.000(0.999-1.002)
	P value	0.707	0.736	0.955	0.525	0.676	0.670

Hypertriglyceridemia, triglyceride ≥ 200 mg/dL; OR, odds ratio; CI, confidence interval; Model I, crude; Model II, age, household income level, education level, and total energy intake adjusted; Total data was additionally adjusted for sex.
